# Supplementary material for: How does explicit reliability guide choices? Effect of trustworthiness on choice and metacognition
Source: PLoS Comput Biol. 2026 Oct 9;22(10):e1014818. doi: 10.1371/journal.pcbi.1014818 (PMC13652329; doi:10.1371/journal.pcbi.1014818)
Supplement: Supplementary file 1 [file pcbi.1014818.s014.docx]

PCOMPBIOL-D-25-02202

Integrating explicit reliability for optimal choices: effect of trustworthiness on decisions and metadecisions

PLOS Computational Biology

Dear Dr. Ota,

Thank you for submitting your manuscript to PLOS Computational Biology. After careful consideration, we feel that it has merit but does not fully meet PLOS Computational Biology's publication criteria as it currently stands. Therefore, we invite you to submit a revised version of the manuscript that addresses the points raised during the review process.

Please submit your revised manuscript by May 17 2026 11:59PM. If you will need more time than this to complete your revisions, please reply to this message or contact the journal office at ploscompbiol@plos.org. When you're ready to submit your revision, log on to <https://www.editorialmanager.com/pcompbiol/> and select the 'Submissions Needing Revision' folder to locate your manuscript file.

Please include the following items when submitting your revised manuscript:

* A letter that responds to each point raised by the editor and reviewer(s). You should upload this letter as a separate file labeled 'Response to Reviewers'. This file does not need to include responses to formatting updates and technical items listed in the 'Journal Requirements' section below.

* A marked-up copy of your manuscript that highlights changes made to the original version. You should upload this as a separate file labeled 'Revised Manuscript with Track Changes'.

* An unmarked version of your revised paper without tracked changes. You should upload this as a separate file labeled 'Manuscript'.

If you would like to make changes to your financial disclosure, competing interests statement, or data availability statement, please make these updates within the submission form at the time of resubmission. Guidelines for resubmitting your figure files are available below the reviewer comments at the end of this letter

We look forward to receiving your revised manuscript.

Kind regards,

Hugues Berry

Section Editor

PLOS Computational Biology

Hugues Berry

Section Editor

PLOS Computational Biology

**Reviewers' comments:**

**Reviewer #1:**

*Ota et al. investigated human decision biases in integrating evidence with explicitly stated reliability (Experiments 1 & 2) and the decision maker’s meta-knowledge about their own biases (Experiment 2). Compared to ideal observers, participants weighted the informative cues more conservatively but meanwhile assigned weights to the non-informative cues; they used the negative cues less efficiently than using the positive ones. Participants’ decisions were best explained by a computational model that assumes both distortion and sequential decay in the use of probabilistic information. A meta-cognitive report after each trial shows that participants were partly aware of their biases.
I think the findings are interesting and the methodology is sound. Most of the conclusions are well supported by the experimental and modeling evidence. This work would make a more significant contribution to the field if the following issues can be addressed.*

**We thank the reviewer for this positive assessment of our manuscript. We found the comments extremely helpful and have implemented several new analyses, which we believe have significantly improved the manuscript. We hope that the manuscript is now suitable for publication.**

*Major concerns:*

*1. In the current winning model, the finding that negative cues (i.e., reliability < 50%) are used less efficiently than positive cues (i.e., reliability > 50%) seems to be explained by probability distortion functions with a crossover point over 50%. I am wondering whether the authors had considered other reasonable explanations. For example, if participants totally ignored the reliability information on a small proportion of trials, they would also show lower efficiency in using negative cues. It would be helpful to have this tested in computational models.*

**We thank the reviewer for raising this point and suggesting an alternative explanation for how participants use evidence from reliably wrong sources. We agree with the reviewer’s prediction that if participants ignored reliability information on a subset of trials that contain reliably wrong information, it could end up demonstrating a lower efficiency of the use of reliability when those are present. However, we believe several aspects of the data and modelling results argue against this interpretation. We nonetheless tested this by including new analyses now reported in the Supplementary Materials.**

**The prediction that, on some trials, participants neglect the reliability of sources would correspond to a distortion parameter alpha approaching zero i.e., the decision is driven solely by the majority colour of the samples, independently of their reliability. Across all experiments, we consistently observe substantial, non-zero weighting of reliability information, indicating that participants did take reliability into account on the vast majority of trials. Importantly, we did not find a lower alpha for experiments that included reliably wrong sources. Rather, we observe that the decrease in performance when reliably wrong sources were present was captured by an increased recency in the evidence accumulation process, rather than a lowering of alpha. This makes it unlikely that, when reliably wrong sources are present, a sizable proportion of trials involve complete disregard of reliability.**

**Nonetheless, we explored alternative model variants in which reliably correct and reliably wrong sources were treated differently: we fitted distinct distortion functions for each of those source categories. Such a model allows us to test specifically the hypothesis that reliably wrong sources are associated with a lower alpha, i.e. less reliance on the reliability of sources. In Experiment 1b, the model with distinct distortion functions showed slightly better predictive performance than the full model, although the difference was small. Importantly however, the resulting alpha value was stronger than in the single distortion model, suggesting that, if anything, there was a stronger sensitivity to reliably wrong sources than correct ones. In contrast, in Experiment 1c, the two models were not different in their predictive abilities. In both cases, the recovered distortion functions were highly similar across model variants, suggesting that the observed asymmetry was better captured by a common distortion process for reliably correct and reliably wrong sources, rather than by a discrete switching or lapse mechanism.**

**Additionally, an account based on trial-wise strategy switching would raise additional assumptions about how and when participants would abandon reliability information, and what alternative strategy they would adopt (e.g., majority counting). As we find that our model captures well the effect of reliably wrong by increased recency in the evidence accumulation process, and we do not find clear behavioural evidence supporting such discrete shifts in strategy, we consider it is not a plausible explanation.**

**To clarify this point, we have added a mention to the supplementary results of a separate fit (Results, lines 435-445) and a discussion of this alternative explanation in the revised manuscript (Discussion, lines 795-799).**

*2. It would be helpful to contrast model predictions with data in the same figure, so that the readers can see which empirical findings can be explained by the models and which cannot.*

**Thank you for this helpful suggestion. We agree that directly comparing model predictions with empirical behaviour improves the interpretability of the modelling results. In the revised manuscript, we have now added the choice behaviour predicted by the model alongside the empirical data, allowing readers to directly assess how well the model captures the observed patterns of behaviour (Results Section lines 431-435). The updated figure shows that the full model reproduced the empirical choice patterns well, including the effects of source reliability and the influence of unreliable and reliably wrong information.**

*3. The following intriguing findings deserve discussion or clarification.*

*(1) How to explain the different sequential weights obtained in different experiments (Figure 4C)? I noticed that the sequential weights were even different for Exp 1a & Exp 2a, which used identical sets of stimuli for decisions.*

**We thank the reviewer for highlighting this point. The sequential weights do indeed differ across experiments, including between Exp. 1a and Exp. 2a, despite the fact that these experiments used identical decision stimuli. The main difference is that Exp. 2a required participants to report on the influence of the sources of a given reliability. One possibility is that this introspective report in itself may have increased attention to the temporal structure of the sequence and thereby altered how evidence was weighted over time. This suggests that sequential weighting is sensitive to broader task context and that metacognition demands might change how participants remember incoming evidence.**

**We have now clarified this point in the Results (lines 524-531), explicitly noting that differences in metacognitive task demands may contribute to the variation in sequential weights observed across experiments.**

*(2) Why did unreliable sources have less effect on behavior for conditions with intermixed positive and negative cues (lines 460–462)?*

**We agree that this pattern deserves clarification. One possible explanation is that conditions with intermixed reliably correct (“positive”) and reliably wrong (“negative”) sources impose greater integration demands, because participants must use the colour two distinct ways across the sequence (respond congruently to the colour for reliably correct sources and incongruently for the reliably wrong sources). Under these circumstances, it is possible that participants pay less attention to the colour per se and rely more on how to interpret it, decreasing the overall effect of mere exposure of one colour and therefore the biasing effect by unreliable sources. Note, however, that the bias by unreliable sources was still observed when all sources were reliably wrong (Exp 2b), suggesting this reframing effect had only a limited scope.**

**We have added this possible explanation to the Discussion (lines 836-842) and emphasize that it should be considered a hypothesis rather than a firm conclusion.**

*4. When analyzing the meta-cognitive report data, the authors had used several clever ways to prove its link with participants’ decisions. I am wonder whether a joint modeling of the decision and meta-cognitive report could be performed.*

**We thank the reviewer for this excellent suggestion. This is, in fact, an issue we had considered previously but initially chose not to pursue in the original submission to keep the modelling framework simpler and to avoid overinterpreting metacognitive reports. We agree, however, with the reviewer that joint modelling of choice and influence reports provides a more principled way to characterise the relationship between the two. We have therefore now extended the manuscript in this direction and we now elaborate on this approach more explicitly in the revised version.**

**We reasoned that the reported subjective influence can be formalised as a counterfactual quantity: estimating the likelihood of making the same choice if the information from the introspected source had been omitted from the evidence sequence and comparing it to the likelihood of making the choice when the information is present. Under this framework, influence ratings effectively reflect the contribution of a given source to the final decision variable. For example, if the choice model assigned a 90% probability to a blue response given the full sequence, but only 60% when the information from the 65% reliable sources was removed, the positive influence of those sources can be quantified as 30%. Conversely, if removing that information increased the probability of the observed choice, the source would be assigned a negative influence. When the probability of the choice was already close to asymptote, however, the additional information would be expected to have little impact, and the corresponding influence estimate would therefore be small.**

**Overall, jointly fitting the choice and influence reports captured well the choice behaviour and the metacognitive reports. The joint model did not lead to different choice-related parameters compared to the choice-only model, indicating that the joint model provided a quantitatively equivalent account of decision behaviour. Crucially, the model also captured well the metacognitive reports. The model reproduced the main patterns observed in the influence ratings, including the monotonic increase in reported influence with the amount of evidence congruent with the choice and the stronger effects for highly reliable sources.**

**We have added a detailed description of this in Methods (lines 1133-1172), Results (lines 635-672, Figure 7) and Discussion (lines 882-898).**

*5. According to their meta-cognitive reports, participants knew their own suboptimal use of non-informative and negative cues (Figure 5). A related question is, did they know their discounting of earlier cues (i.e., the sequential weight)?*

**Thank you for this interesting suggestion. We agree that an important related question is whether participants also have metacognitive access to their sequential weighting of evidence, such as the discounting of earlier cues. This is indeed a question we have already asked ourselves and we have started designing a dedicated experiment to address it more directly.**

**Our view, however, is that this question is not easily resolved within the present dataset and would require a substantially different design. In particular, one would need to assess not only the objective contribution of each sample as a function of its serial position, but also participants’ memory for individual samples and their subjective estimate of each sample’s influence on the final choice (for example, the perceived influence of the first versus last sample). Because this requires careful dissociation of memory, evidence integration and introspective access at the level of individual samples, we believe it is better suited to a separate study specifically designed for that purpose, rather than being incorporated only partially into the present article. We have nonetheless added this point to the Discussion (lines 927-932) as an important direction for future work.**

Minor issues
The Results section should be self-contained since it appears ahead of the Methods. For example, please describe the number of participants in each experiment at the beginning of the Results.

**We thank the reviewer for suggesting this improvement. We have revised the Results section to make the experimental design clear without needing to refer immediately to the Methods section. We added key methodological details, including the number of participants and trials in each experiment and the sequential structure of the task (lines 192-212).**

Figure 1B: Please describe the stimulus sequence that is illustrated in the figure.

**We have updated the figure legend (lines 244-250) to make it clearer that it corresponds to the presented sequence.**

Figure 2A: the gray and black colors are difficult to tell apart.

**Thanks for noting this lack of readability. We have updated the colours in Figure 2A to make symbols visually distinguishable.**

lines 263: typo 69.4.2%

**Thanks for noticing. The typo has now been corrected.**

lines 319-324: Why was the beta sometimes positive and sometimes negative when similar effects in different experiments were described?

**We thank the reviewer for this point. The sign of the beta reflects the direction of the evidence, not its informativeness. For reliably wrong sources (below 50% reliability), the optimal observer should infer the opposite of the predicted colour. Their effect, therefore, has the opposite sign to that of reliably right sources, even though it is equally strong. Thus, the sign change reflects reversed directional evidence, whereas the similar magnitude reflects equal informativeness. We have clarified this in the revised text (lines 326-329).**

Supplementary Figures 1 & 2: please provide the correlation coefficients between the fitted and simulated parameters.

**We have updated Supplementary Figures 1&2 (now labelled as Supplementary Figures 2&3) and added the correlation coefficients. As shown in these figures, we find strong correlations between the fitted and simulated parameters. While generating this figure, we detected a minor error in the parameter recovery procedure concerning the specification of the prior distribution for each parameter. Although this did not affect the results, it has now been corrected. We also changed the figure of the sequential weights at the individual level to show a recovery of sequential weights from the first sample to the fifth sample, rather than collapsing weights across samples.**

lines 543: The notations for the quadratic model does not need the “*”.

**Thanks for noticing this typo. We have removed the asterisk.**

**Reviewer #2**:

*In this study, Ota and colleagues present an inference task where participants are provided with explicit cues on the reliability of information relevant to make a decision. The reliability of the information is described as percentages without ambiguity. The authors examine how human participants combine six samples of information and their reliability in a 2AFC between color categories. Participants take into account the information and their reliability yet in a distorted manner. The manipulation that when reliability is below 50% it means evidence for the alternative color category, not the color indicated, is more difficult for participants to take into account.

The paper is well written and the figures are didactic and appropriate. The authors should also be commended on running five variants of their experiment to get a sense of the strength of the effects, and further study subjective reports related to participants' choices.

I have two main comments about the interpretation of the key manipulation which I explain below, and I think the computational modeling analysis needs major additional checks for being able to support the claims provided.*

**We thank the reviewer for this thoughtful and constructive assessment of our manuscript. We are grateful for the positive evaluation of the writing, figures, and experimental scope, as well as for the careful attention given to both the conceptual interpretation and the computational modelling. We found these comments extremely helpful. In response, we have substantially revised the manuscript, clarified the interpretation of the key manipulation and added several new analyses and checks for the computational modelling. We believe these changes have significantly strengthened the paper and we hope that the revised manuscript is now suitable for publication.**

*Conceptual comments*

*1- Even if the experimenters explicitly provide the reliability to participants on the screen, one needs to trust the source. In other words, the reliability of that reliability information. I would like to challenge the assumption that participants simply trust the experimenters' information ("by an objective external source"). Therefore, I do not know if I would characterise the deviations found as suboptimality, especially in light of the reasonable awareness participants had in the making of their choices. Throughout the paper I was wondering to what extent the findings are related to the design itself: participants may not entirely believe the experimenters about the trustworthiness of the explicit cues provided and therefore do not treat it objectively. Is the key contrast between explicit vs. implicit or unknown, or between trustworthy and untrustworthy?*

**We thank the reviewer for this important conceptual point. We agree that explicitly providing a reliability value does not guarantee that participants treat it as fully trustworthy.**

**Indeed, such criticism extends to any study that provides probabilities to participants: even if the experimenter suggests a 65% likelihood of a reward, participants may still discount that information if they are uncertain about how reliable or well-calibrated such a prediction really is. More broadly, this means that the question of probability distortion and perception of probability cannot be fully separated from questions about how much participants trust numerical information (and the source of those numbers) to reflect the true structure of the world.**

**Our use of the term *suboptimality* here is only relative to a normative benchmark, namely an ideal observer who fully trusts and uses the stated reliabilities. We then characterise deviations from this benchmark, but to some extent, we cannot fully identify their cause. In that sense, it does not allow us to dissociate a distorted subjective perception of likelihood from the lack of trust in that information. In that sense, *suboptimality* does not necessarily imply irrationality in a broader psychological sense. Indeed, a large body of literature suggests that probability distortion reflects a form of bounded rationality, in particular Zhang & Maloney (2020), which has been an important inspiration for this work.**

**We have now added a passage to the Discussion (lines 852-862) to acknowledge this point more clearly and to note that some of the observed deviation from the ideal-observer benchmark may reflect partial discounting of the stated reliabilities, rather than a failure to use reliability information per se. We have also removed the use of the terms “optimal” and “suboptimal” throughout the text when it was not used directly in comparison to the Bayesian observer or could be misinterpreted, including in the title of the manuscript.**

2- *I wonder if participants really interpreted the below 50% percentages indicating reliably wrong or evidence in favor of the alternative choice. Rather, in intuitive units, 0% would indicate no evidence in favour of either color. It seems counterintuitive that given the color of the square the evidence is in favour of the other square. I struggle to find an example of real-life information delivered in such a manner. I think this is why participants do not integrate it as well as the reliably right information, because they have this symmetrical operation to do opposite of the colour presented. I appreciate that experimenters have delivered careful instructions about the meaning of these percentages, but the authors themselves acknowledge that cognitive load may influence the results: more cognitive load is required when having to convert reliably wrong information for choice, hence participants do less well.*

**We thank the reviewer for this thoughtful comment. We agree that information below 50% reliability is less intuitive, because it must be interpreted in the opposite direction to the colour that is explicitly displayed. This likely makes such information more demanding to use than reliably correct information, even when participants are carefully instructed about its meaning.**

**At the same time, we do not think this format is entirely disconnected from real-world cognition. Humans naturally represent the possibility that others may be deceptive, lying being a familiar part of social interactions. In everyday life, people often encounter sources that are known to be consistently biased, deceptive, or predictably wrong, such as an overly promotional salesperson, a highly partisan commentator or a habitual liar repeatedly denying responsibility. In such cases, the source can remain informative, but its value lies in the opposite direction to its literal content. Importantly, this does not require the source to be wrong on every single occasion. Rather, it is enough that, on balance, its claims are more safely interpreted as evidence for the alternative state of the world.**

**We nevertheless agree with the reviewer that this inverse mapping is likely more cognitively demanding than simply following a reliably correct cue. Thus, any asymmetry between the use of reliably right and reliably wrong information may partly reflect this additional processing demand, rather than a failure to understand the stated reliabilities themselves. We have now clarified this point in the Introduction (lines 151-162) and the Discussion (lines 721-733).**

*Computational modeling comments

3 - The study needs a model recovery: there are four alternative models which is a good aspect but no test of model identifiability has been performed (please see Wilson and Collins 2019 for guidance).*

**We thank the reviewer for this important suggestion. We agree that demonstrating model identifiability is essential when comparing several candidate models. Following this recommendation, we performed a full model recovery analysis across the four model variants considered in the manuscript: the Bayesian model, the sequential weights model, the reliability distortion model, and the full model.**

**Overall, model recovery was very good, with a mean recovery accuracy of 98.4%. The inversion matrix was strongly near-diagonal, indicating that the fitting procedure was generally able to recover the true generating model reliably. The lowest recovery rate was observed for the Bayesian model (70%). Importantly, however, the converse pattern was highly reassuring: whenever the Bayesian model was selected as the best-fitting model, it corresponded to the true generating model in 100% of cases. More generally, across models, when a model was identified as the best-fitting one, it had a greater than 98% probability of being the true generating model.**

**These results indicate that the four models are well distinguishable and that the model-comparison results reported in the manuscript are unlikely to reflect poor identifiability. We have now added this model recovery analysis to the revised manuscript in the Results section (lines 396-403) and the procedure for model recovery in the Methods section (lines 1276-1307).**

*4- Given that the winning model has both source of distortions, it seems incorrect to me to interpret that the model with distortion alone being better than the sequential weights alone (both losing).*

**We thank the reviewer for this comment and for noticing this inaccuracy in the text. We agree that the model with distortion alone is not comparable with the model with the sequential weights alone. The expected log pointwise predictive accuracy (elpd) was compared between the best model and alternative models. Therefore, the model inference should be made relative to only the best model. We revised the Results section to clarify this point (lines 419-430).**

*5 - The section reporting model comparison presents no statistical values or any quantitative measurement; from Fig4A it seems that the second best fitting model is very close so it would be good to know by how much. In particular, the winning model has the most degree of freedom (I appreciate that the authors explain in the Methods that their chosen method do not take into account df)*

**We thank the reviewer for this helpful suggestion. We agree that the model-comparison results should be reported more quantitatively, especially where the top-ranked models appear visually close in Figure 4A. To address this, we have now added the relevant leave-one-out expected predictive density differences between the best-fitting and second-best-fitting models, directly in the Results (lines 419-430). This makes the strength of the model preference more explicit and easier to evaluate.**

**We also appreciate the reviewer’s concern that the winning model has the largest number of free parameters. We now clarify more explicitly that our model comparison was based on leave-one-out expected predictive density, which assesses out-of-sample predictive performance rather than raw in-sample fit (lines 1203-1205). As such, the comparison does not simply favour the most flexible model because it has more parameters but instead evaluates whether the additional complexity improves generalisation to unseen data. We have revised the text accordingly to make this rationale clearer.**

*6 - The parameter recovery only show recovered parameters, but do not tell us about their identifiability (please see Wilson and Collins 2019). It would be important to know how much parameters correlate between themselves vs are independent. The group values presented show that parameter estimation is quite heavily biased for the sequential weights. The fitting procedure overestimate small weights and underestimate large weights so it is quite problematic to interpret the weights that participants give to each piece of information in the sequence.*

**We thank the reviewer for this important point. We agree that parameter recovery should not be evaluated only in terms of correspondence between true and recovered values, but also in terms of parameter identifiability, including the extent to which parameters can be dissociated from one another.**

**To address this concern, we have now added a correlation matrix of the individual-level parameter estimates (Supplementary Figure 11). Overall, correlations between parameters were weak, indicating limited trade-off between parameters and therefore good identifiability within the model. This provides additional support that the fitted parameters capture distinct aspects of behaviour rather than reflecting strong mutual dependence.**

**Regarding the reviewer’s concern about bias in the recovery of the sequential weights, we agree that this is an important issue when interpreting the absolute magnitude of these parameters. We now clarify this point more explicitly in the revised manuscript (Results lines 419-430). In particular, while the recovery results indicate that sequential weights should be interpreted with some caution at the level of their exact numerical value, the overall pattern of recovery and the weak parameter correlations suggest that the fitting procedure is nonetheless able to distinguish the contribution of sequential weighting from that of the other model components. We have revised the text accordingly and now discuss these limitations more explicitly (Results lines 539-541 and Discussion lines 923-924).**

Other comments

*7 - Participants receive feedback after each choice. Whether any learning across trials occurs, or any changes in strategy for integrating information across samples is not at all adressed (trials are analysed independently). Is it possible that participants get better (more objective) at integrating the presented probabilities for their choice after some practice with the task?*

**We thank the reviewer for this important point. We agree that trial-by-trial feedback could lead to learning across the experiment, including a progressively better use of the stated reliabilities or changes in integration strategy. Our main analyses treated trials independently and were therefore not designed to characterise such across-trial adaptation.**

**We agree, however, that participants may become more objective or change the way they perceive reliability with experience. Addressing this question properly requires analyses and experimental manipulations specifically designed to track learning over time, which we are pursuing in ongoing work and that we aim to publish in another paper. We now acknowledge this limitation more explicitly in the Discussion (lines 763-770).**

*8 - In the logistic regression analysis (Fig supp 4), the reliabilities were recoded rather than using the true percentages: could the authors justify this analysis choice?*

**On each trial, one of the three reliability levels was chosen to evaluate the influence of that reliability on the choices participants made. Treating the introspected reliability levels as a continuous variable rather than a categorical variable (factor) forces a linear interpolation on the slopes of the introspective report across the three levels. Rather, we treated the reliability levels as a factor, which allowed us to estimate the slopes of the introspective reports in reaction to the net number of samples (evidence for choice), separately for each of the three levels of introspected reliability. We clarified this point in the Methods (ln 1380-1386).**

*Minor comments

9 - The metacognitive question is not asking participants how confident in their color choice they are, but is one of credit assignment: 'how did the 65% reliable squares influence your decision?". The authors interpret the response as participants having 'a stronger sense of being influenced', whereas it seems to me that they are not influenced as much as just telling the experimenters what piece of information they used. I think it could be discussed, not all subjective judgements are necessarily metacognitive.*

**We thank the reviewer for this important point. We agree that the question in Experiment 2 is not a standard confidence judgement about the correctness of the colour choice, but rather a report about the perceived influence of a given source on that choice. This comment also converges with Reviewer 1’s suggestion that choice and influence should be modelled jointly, and we now elaborate this issue much more explicitly in the revised manuscript.**

**More specifically, we now clarify that influence can be related to confidence under a counterfactual interpretation. If confidence is understood as reflecting the strength of evidence supporting the chosen option, then perceived influence can be formalised as the change in the likelihood of making the same choice when a given piece of evidence is included versus omitted. In other words, the influence judgment can be interpreted as a counterfactual question: how likely would I have been to make the same choice if this source had not been presented?**

**We now incorporate this interpretation into our model and show that it accounts well for the reported influence ratings. Importantly, this counterfactual formulation is mathematically equivalent to quantifying the amount of evidence contributed by the introspected source to the final choice. In that sense, the influence report is not merely an arbitrary verbal attribution, but can be linked to a principled quantity in the decision process.**

**We agree, however, that whether such a judgement should be considered genuinely metacognitive remains an important conceptual question. To some extent, the issue is whether access to the quantity of information supporting one’s choice should itself count as metacognitive. We now discuss this question more carefully in the revised Discussion (lines 888-898) and clarify the precise sense in which we use this interpretation.**

*10 - In the introduction, conceptually 'trust', 'confidence', and 'precision' of beliefs are used interchangeably, whereas these notions refer to quite different quantities and different frameworks in the literature.*

**We thank the reviewer for noting this inconsistency. We agree that *trust*, *confidence*, and *precision* refer to distinct constructs and should not be treated as interchangeable without clarification. At the same time, these terms are often used in partly overlapping ways across different literatures, which may have contributed to the imprecision in our original phrasing. For this reason, we have kept the original terminology where it reflects the standard usage within each literature.**

*11 - Likewise the terminology shifts between 'reliability', 'opinions", "voting", whereas the task is perceptual and not framed as social, I would suggest to keep the same terms for clarity.*

**We thank the reviewer for this helpful suggestion. We agree that greater terminological consistency improves clarity, especially given that the task is perceptual rather than explicitly social in framing. We have therefore revised the manuscript to use more consistent terminology throughout, favouring terms appropriate to perceptual decision-making. In particular, we now refer to each source’s colour as “prediction” about the correct colour, rather than using terms such as “opinion” or “voting.”**

*12 - In my opinion, the Discussion parts related to implications for the processing of onlines fake news is besides the present topic since even when explicit information is provided, we do not know whether agents trust it (rather than assuming agents trust it and ask if they do not accurately process it).*

**We thank the reviewer for this important point. We agree that the link to online misinformation should be drawn more cautiously. In particular, the present paradigm does not directly address the question of trustworthiness in reliability information itself. We have therefore toned down this part of the Discussion (lines 721-733 and 956-961) and now present the relation to misinformation as a broader possible context of relevance, rather than as a direct implication of the present findings.**

*13 - On the Discussion about distorted probabilities, I think a reason the authors do not find the classic Tversky and Kahneman shape relates to the description-experience gap.*

**We thank the reviewer for this insightful comment. We agree that the description–experience gap could be important when interpreting the form of the probability distortion observed here. We already mentioned this point in the Discussion but have now strengthened this argument.**

**More specifically, classic Tversky and Kahneman probability weighting functions were derived primarily in tasks involving explicitly described probabilities, often in the context of single risky choices about gains and losses. By contrast, in our paradigm, although reliability values were explicitly displayed, participants also accumulated evidence sequentially across samples and received trial-by-trial feedback. In that respect, the task does not map cleanly onto a standard description-based setting but may share some features with experience-based decisions. This may help explain why the distortion pattern we observed does not fully match the classic inverse-S shape.**

**We also now clarify that this is not the only possible explanation. The apparent S-shape may partly reflect modelling assumptions, because the sensitivity parameter cannot be estimated independently of the inverse temperature of the decision rule. Alternatively, it may reflect a more specific property of source-reliability judgements: evaluating whether a source is informative or trustworthy may engage processes that differ from those involved in judging the likelihood of an outcome.**

**We have revised the Discussion accordingly (lines 750-779) to make clear that the discrepancy with the classic Tversky and Kahneman pattern may reflect the hybrid description–experience nature of the task, while also acknowledging other possible explanations and emphasizing that the present data establish distorted use of stated reliabilities, but not the precise functional form of this distortion.**

*14 - The comparison of sequential weights between Experiments 2a and 2b is attributed to experimental differences whereas it could also simply be a different group of participants.*

**We thank the reviewer for noting this. We agree that, because Experiments 2a and 2b were conducted in different groups of participants, differences in sequential weights cannot be attributed unambiguously to the experimental manipulation alone. Group differences may also have contributed to this effect. We have therefore revised the text (lines 524-531) to make this interpretation more cautious and to avoid implying a purely experimental cause.**
