# Supplementary material for: How does explicit reliability guide choices? Effect of trustworthiness on choice and metacognition
Source: PLoS Comput Biol. 2026 Oct 9;22(10):e1014818. doi: 10.1371/journal.pcbi.1014818 (PMC13652329; doi:10.1371/journal.pcbi.1014818)
Supplement: Supplementary file 2 [file pcbi.1014818.s015.docx]

**Comments to the Authors:**

**Please note here if the review is uploaded as an attachment.**

Reviewer #1: I'd like to thank the authors for addressing all my previous questions.

Reviewer #2: I have now carefully read the authors' replies to earlier comments, and I can see that the manuscript has strengthened. There is one major point that remains not fully clarified is the computational model analysis, which I explain below (previous comment 4).

Supplementary Fig 12 indicates correlations between best fitting parameters are all relatively low, so the authors write that parameters are independent. In contrast, the authors elsewhere also write that parameters are not independent: "The apparent S-shape may partly reflect modelling assumptions, because the sensitivity parameter cannot be estimated independently of the inverse temperature of the decision rule."

We are also told that the softmax temperature parameter is fixed not fitted, due to issues of identifiability; fixing this parameter is actually a strong assumption, which impacts the rest of the parameters interpretation for instance the weights. This assumption should be made more prominent, not only in the Methods section, for readers to properly interpret the findings.

We thank the reviewer for raising this important point. We agree that the sensitivity parameter cannot be identified independently of the inverse temperature of the softmax. This is precisely why the inverse temperature was fixed to one throughout the analysis. Our intention was never to claim that the absolute magnitude of the recovered distortion parameters is identifiable independently of this normalisation. Rather, our goal was to characterise behaviour under a standard normalisation, while emphasising that the sign bias by unreliable sources and the effect of the ordering of the information samples do not depend on this choice. We realise that this distinction may not have been sufficiently salient for readers, and we have therefore strengthened the corresponding explanations in the Results.

*Results (line 383-388)*

*The inverse temperature of the softmax was fixed to 1 for parameter identifiability. Consequently, all recovered subjective reliability values should be interpreted under this normalization; qualitative conclusions (such as the sign of the presentation bias by unreliable sources and the effect of the relative ordering of the information samplesreliability levels) are invariant to this choice , so the recovered magnitudes of the distortion parameters are conditional on this choice, although the sign of the presentation bias and the relative ordering of reliability levels are not (see Methods). Under this normalisation, the logistic choice rule implies that the probability of choosing blue equals the participant’s subjective posterior probability that the stimulus is blue.*

The authors acknowledge in the Methods section that the other parameter observed differences could in principle reflect a difference in choice stochasticity, but then the whole paper does not shy away from interpreting the other parameter distortions and make conclusions on over/underweighting of information.

We agree that the recovered subjective reliabilities should be interpreted under the fixed-temperature normalization rather than as uniquely identifiable psychological quantities. This conditional interpretation was already stated in the Results (line 483-491). To avoid any ambiguity, we have now reinforced this point in the caption of Figure 4 and the Discussion.

*Figure 4. Results of computational analysis.*

*(A) Expected log pointwise predictive density (elpd) denotes the out-of-sample predictive accuracy of the model explaining the choice data. The elpd difference between the best model (i.e., full model) and alternative models is plotted. Error bar denotes one standard error of the difference in elpd. The full model, which had distortion of reliability and sequential weights on information sources, accounted for the data best. (B) Recovered subjective reliability percentage from the full model under the fixed-temperature normalisation (θ = 1 ). (C) The sequential weights recovered from the full model.*

*Discussion (line 751-769)*

*We found that participants behaved as if they underestimated small reliability percentages (reliably wrong) and overestimated medium-to-large reliability percentages (reliably correct), resembling an “S-shaped” function. Three broad explanations could account for this pattern, opposite to the canonical inverted-S weighting reported above. A first, if somewhat less theoretically informative, possibility is that the apparent S-shape may result from the way choice stochasticity was captured in the model. Because the sensitivity parameter (a) cannot be estimated separately from the inverse temperature of the decision rule (θ, see Methods). Accordingly, the precise curvature of the recovered distortion function should not be interpreted independently of the normalization imposed on the softmax. Therefore, the recovered magnitude of a, which determines if there is an amplification (S-shape, a > 1) or compression (inverse-S, a < 1) of the probability values, is conditional on the value of θ assumed. As we assumed here θ = 1, the distortion could become inverse-S if the true inverse temperature value of θ was substantially larger, exceeding the recovered sensitivity (a) itself (more than 1.7 to 3, depending on the experiment). In that sense, the distortion recovered here might have absorbed sources of variability not entirely due to distortions of reliability. Determining if that is the case would require asking participants to report continuous probability estimates, rather than relying on choices alone, with the caveat that such reports would introduce new modelling challenges on how subjective probabilities are generated, transformed and mapped onto the response scale.*

Model recovery: the authors have filtered out fits that did not converge. How many were filtered out besides the 64 fits selected? Could the authors explain how can we ensure that issues of convergence did not affect the fits of participants' data?

In the choice model, none were excluded. In the joint model, 63/768 fits were excluded. The excluded fits were predominantly associated with uniform weight profiles and low sensitivity values, consistent with the expectation that individual sequential weights are only identifiable when they differ across positions in the sequence. Importantly, none of the fits to the actual participant data were excluded due to convergence issues. Thus, convergence problem did not affect the participant-level parameter estimates reported in the manuscript. We added this explanation as follows.

*Line 1267-1279: Each synthetic dataset was then refitted with the joint model described in the model fitting procedure using a MCMC sampling. Fits were excluded from the recovery summaries if they showed poor convergence, defined as a maximum R-hat ≥1.1 or 50 or more divergent transitions. The excluded fits were predominantly associated with uniform weight profiles and low sensitivity values, consistent with the expectation that individual sequential weights are only identifiable when they differ across positions in the sequence. This resulted in 705 (out of 768) fits being retained for the final analysis. Since none of the fits to the actual participant data were excluded due to convergence issues, this exclusion did not affect the participant-level parameter estimates. Parameter recovery was visualised by plotting recovered estimates against the true generating values, separately for group-level parameter means (Supplementary Figure 8) and individual-level parameters (Supplementary Figure 9). In addition, parameter dependence was visualised by plotting the pairwise correlation matrix of the posterior means of the individual-level parameters (Supplementary Figure 12).*

p108 the fitted weights are underestimated by the fitting procedure, not overestimated, as per fig supp 4.

There may be confusion in the original sentence. The panels C-D in Supplementary Figure 3 show that, at the individual level of the parameter recovery, the fitted weights were overestimated when the true generating values were less than 1, while the fitted weights were underestimated when the true generating values were larger than 1. We have corrected the corresponding sentence as follows.

[*Line 400-404: P*](https://docs.google.com/document/d/1_GdEwYxwenebZ85QJ_4nWmesumtIPRoG/edit#bookmark=id.xaiu0of0c5a7)*arameter recovery also confirmed that recovery of group-level parameters and individual-level parameters was robust (Supplementary Figure 2 & Supplementary Figure 3; see Methods),* *although sequential weights at the individual level tended to be overestimated when the true generating value was below 1 and overestimated when it was above 1.*

Next time, please print the quotes in the revised manuscript for each comment as is standardly done. It was time consuming to scroll back and forth to find edits in-between each response.

We apologise for the inconvenience. We have now included all the modified text in the present response.

In your final version please check the supplementary figures in the revised manuscript, many were mismatched with the legend or missing.

We have thoroughly checked all supplementary figures, their corresponding legends, and the locations where they are cited in the revised manuscript. We have ensured that all figure references, legends, and accompanying text correctly correspond to the relevant supplementary figures.
