# Supplementary material for: How does explicit reliability guide choices? Effect of trustworthiness on choice and metacognition
Source: PLoS Comput Biol. 2026 Oct 9;22(10):e1014818. doi: 10.1371/journal.pcbi.1014818 (PMC13652329; doi:10.1371/journal.pcbi.1014818)
Supplement: Peer review history

# Peer review: Decision Letter 0

**DOI:** 10.1371/journal.pcbi.1014818.r001

**Authors:** Hugues Berry

17 Mar 2026

Integrating explicit reliability for optimal choices: effect of trustworthiness on decisions and metadecisions

PLOS Computational Biology

Dear Dr. Ota,

Thank you for submitting your manuscript to PLOS Computational Biology. After careful consideration, we feel that it has merit but does not fully meet PLOS Computational Biology’s publication criteria as it currently stands. Therefore, we invite you to submit a revised version of the manuscript that addresses the points raised during the review process.

Please submit your revised manuscript by May 17 2026 11:59PM. If you will need more time than this to complete your revisions, please reply to this message or contact the journal office at ploscompbiol@plos.org. When you're ready to submit your revision, log on to https://www.editorialmanager.com/pcompbiol/ and select the 'Submissions Needing Revision' folder to locate your manuscript file.

Please include the following items when submitting your revised manuscript:

* A letter that responds to each point raised by the editor and reviewer(s). You should upload this letter as a separate file labeled 'Response to Reviewers'. This file does not need to include responses to formatting updates and technical items listed in the 'Journal Requirements' section below.

* A marked-up copy of your manuscript that highlights changes made to the original version. You should upload this as a separate file labeled 'Revised Manuscript with Track Changes'.

* An unmarked version of your revised paper without tracked changes. You should upload this as a separate file labeled 'Manuscript'.

If you would like to make changes to your financial disclosure, competing interests statement, or data availability statement, please make these updates within the submission form at the time of resubmission. Guidelines for resubmitting your figure files are available below the reviewer comments at the end of this letter

We look forward to receiving your revised manuscript.

Kind regards,

Hugues Berry

Section Editor

PLOS Computational Biology

Hugues Berry

Section Editor

PLOS Computational Biology

**Journal Requirements:**

1) Please ensure that the CRediT author contributions listed for every co-author are completed accurately and in full.

At this stage, the following Authors/Authors require contributions: Keiji Ota, Patrick Haggard, Thibault Gajdos Preuss, Lucie Charles, and Anthony Ciston. Please ensure that the full contributions of each author are acknowledged in the "Add/Edit/Remove Authors" section of our submission form.

The list of CRediT author contributions may be found here: https://journals.plos.org/ploscompbiol/s/authorship#loc-author-contributions

2) Thank you for including an Ethics Statement for your study.

i) Please state whether the consent obtained is verbal or written.

3) Please upload all main figures as separate Figure files in .tif or .eps format. For more information about how to convert and format your figure files please see our guidelines:

https://journals.plos.org/ploscompbiol/s/figures

4) We notice that your supplementary Figures are included in the manuscript file. Please remove them and upload them with the file type 'Supporting Information'. Please ensure that each Supporting Information file has a legend listed in the manuscript after the references list.

5) Some material included in your submission may be copyrighted. According to PLOSu2019s copyright policy, authors who use figures or other material (e.g., graphics, clipart, maps) from another author or copyright holder must demonstrate or obtain permission to publish this material under the Creative Commons Attribution 4.0 International (CC BY 4.0) License used by PLOS journals. Please closely review the details of PLOSu2019s copyright requirements here: PLOS Licenses and Copyright. If you need to request permissions from a copyright holder, you may use PLOS's Copyright Content Permission form.

Please respond directly to this email and provide any known details concerning your material's license terms and permissions required for reuse, even if you have not yet obtained copyright permissions or are unsure of your material's copyright compatibility. Once you have responded and addressed all other outstanding technical requirements, you may resubmit your manuscript within Editorial Manager.

Potential Copyright Issues:

i) Figures 1, and 3. Please confirm whether you drew the images / clip-art within the figure panels by hand. If you did not draw the images, please provide (a) a link to the source of the images or icons and their license / terms of use; or (b) written permission from the copyright holder to publish the images or icons under our CC BY 4.0 license. Alternatively, you may replace the images with open source alternatives. See these open source resources you may use to replace images / clip-art:

- https://commons.wikimedia.org

- https://openclipart.org/.

6) Please amend your detailed Financial Disclosure statement. This is published with the article. It must therefore be completed in full sentences and contain the exact wording you wish to be published.

1) State what role the funders took in the study. If the funders had no role in your study, please state: "The funders had no role in study design, data collection and analysis, decision to publish, or preparation of the manuscript.".

Note: If the reviewer comments include a recommendation to cite specific previously published works, please review and evaluate these publications to determine whether they are relevant and should be cited. There is no requirement to cite these works unless the editor has indicated otherwise.

**Reviewers' comments:**

Reviewer's Responses to Questions

Reviewer #1: Ota et al. investigated human decision biases in integrating evidence with explicitly stated reliability (Experiments 1 & 2) and the decision maker’s meta-knowledge about their own biases (Experiment 2). Compared to ideal observers, participants weighted the informative cues more conservatively but meanwhile assigned weights to the non-informative cues; they used the negative cues less efficiently than using the positive ones. Participants’ decisions were best explained by a computational model that assumes both distortion and sequential decay in the use of probabilistic information. A meta-cognitive report after each trial shows that participants were partly aware of their biases.

I think the findings are interesting and the methodology is sound. Most of the conclusions are well supported by the experimental and modeling evidence. This work would make a more significant contribution to the field if the following issues can be addressed.

Major concerns:

1. In the current winning model, the finding that negative cues (i.e., reliability < 50%) are used less efficiently than positive cues (i.e., reliability > 50%) seems to be explained by probability distortion functions with a crossover point over 50%. I am wondering whether the authors had considered other reasonable explanations. For example, if participants totally ignored the reliability information on a small proportion of trials, they would also show lower efficiency in using negative cues. It would be helpful to have this tested in computational models.

2. It would be helpful to contrast model predictions with data in the same figure, so that the readers can see which empirical findings can be explained by the models and which cannot.

3. The following intriguing findings deserve discussion or clarification. (1) How to explain the different sequential weights obtained in different experiments (Figure 4C)? I noticed that the sequential weights were even different for Exp 1a & Exp 2a, which used identical sets of stimuli for decisions. (2) Why did unreliable sources have less effect on behavior for conditions with intermixed positive and negative cues (lines 460–462)?

4. When analyzing the meta-cognitive report data, the authors had used several clever ways to prove its link with participants’ decisions. I am wonder whether a joint modeling of the decision and meta-cognitive report could be performed.

5. According to their meta-cognitive reports, participants knew their own suboptimal use of non-informative and negative cues (Figure 5). A related question is, did they know their discounting of earlier cues (i.e., the sequential weight)?

Minor issues:

The Results section should be self-contained since it appears ahead of the Methods. For example, please describe the number of participants in each experiment at the beginning of the Results.

Figure 1B: Please describe the stimulus sequence that is illustrated in the figure.

Figure 2A: the gray and black colors are difficult to tell apart.

Line 263: typo 69.4.2%

Line 319-324: Why was the beta sometimes positive and sometimes negative when similar effects in different experiments were described?

Supplementary Figures 1 & 2: please provide the correlation coefficients between the fitted and simulated parameters.

Line 543: The notations for the quadratic model does not need the “*”.

Reviewer #2: In this study, Ota and colleagues present an inference task where participants are provided with explicit cues on the reliability of information relevant to make a decision. The reliability of the information is described as percentages without ambiguity. The authors examine how human participants combine six samples of information and their reliability in a 2AFC between color categories. Participants take into account the information and their reliability yet in a distorted manner. The manipulation that when reliability is below 50% it means evidence for the alternative color category, not the color indicated, is more difficult for participants to take into account.

The paper is well written and the figures are didactic and appropriate. The authors should also be commended on running five variants of their experiment to get a sense of the strength of the effects, and further study subjective reports related to participants' choices.

I have two main comments about the interpretation of the key manipulation which I explain below, and I think the computational modeling analysis needs major additional checks for being able to support the claims provided.

Conceptual comments

Even if the experimenters explicitly provide the reliability to participants on the screen, one needs to trust the source. In other words, the reliability of that reliability information. I would like to challenge the assumption that participants simply trust the experimenters' information ("by an objective external source"). Therefore, I do not know if I would characterise the deviations found as suboptimality, especially in light of the reasonable awareness participants had in the making of their choices. Throughout the paper I was wondering to what extent the findings are related to the design itself: participants may not entirely believe the experimenters about the trustworthiness of the explicit cues provided and therefore do not treat it objectively. Is the key contrast between explicit vs. implicit or unknown, or between trustworthy and untrustworthy?

I wonder if participants really interpreted the below 50% percentages indicating reliably wrong or evidence in favor of the alternative choice. Rather, in intuitive units, 0% would indicate no evidence in favour of either color. It seems counterintuitive that given the color of the square the evidence is in favour of the other square. I struggle to find an example of real-life information delivered in such a manner. I think this is why participants do not integrate it as well as the reliably right information, because they have this symmetrical operation to do opposite of the colour presented. I appreciate that experimenters have delivered careful instructions about the meaning of these percentages, but the authors themselves acknowledge that cognitive load may influence the results: more cognitive load is required when having to convert reliably wrong information for choice, hence participants do less well.

Computational modeling comments

The study needs a model recovery: there are four alternative models which is a good aspect but no test of model identifiability has been performed (please see Wilson and Collins 2019 for guidance).

Given that the winning model has both source of distortions, it seems incorrect to me to interpret that the model with distortion alone being better than the sequential weights alone (both losing).

The section reporting model comparison presents no statistical values or any quantitative measurement; from Fig4A it seems that the second best fitting model is very close so it would be good to know by how much. In particular, the winning model has the most degree of freedom (I appreciate that the authors explain in the Methods that their chosen method do not take into account df)

The parameter recovery only show recovered parameters, but do not tell us about their identifiability (please see Wilson and Collins 2019). It would be important to know how much parameters correlate between themselves vs are independent. The group values presented show that parameter estimation is quite heavily biased for the sequential weights. The fitting procedure overestimate small weights and underestimate large weights so it is quite problematic to interpret the weights that participants give to each piece of information in the sequence.

Other comments

Participants receive feedback after each choice. Whether any learning across trials occurs, or any changes in strategy for integrating information across samples is not at all adressed (trials are analysed independently). Is it possible that participants get better (more objective) at integrating the presented probabilities for their choice after some practice with the task?

In the logistic regression analysis (Fig supp 4), the reliabilities were recoded rather than using the true percentages: could the authors justify this analysis choice?

Minor comments

The metacognitive question is not asking participants how confident in their color choice they are, but is one of credit assignment: 'how did the 65% reliable squares influence your decision?". The authors interpret the response as participants having 'a stronger sense of being influenced', whereas it seems to me that they are not influenced as much as just telling the experimenters what piece of information they used. I think it could be discussed, not all subjective judgements are necessarily metacognitive.

In the introduction, conceptually 'trust', 'confidence', and 'precision' of beliefs are used interchangeably, whereas these notions refer to quite different quantities and different frameworks in the literature.

Likewise the terminology shifts between 'reliability', 'opinions", "voting", whereas the task is perceptual and not framed as social, I would suggest to keep the same terms for clarity.

In my opinion, the Discussion parts related to implications for the processing of online fake news is besides the present topic since even when explicit information is provided, we do not know whether agents trust it (rather than assuming agents trust it and ask if they do not accurately process it).

On the Discussion about distorted probabilities, I think a reason the authors do not find the classic Tversky and Kahneman shape relates to the description-experience gap.

The comparison of sequential weights between Experiments 2a and 2b is attributed to experimental differences whereas it could also simply be a different group of participants.

**********

**Have the authors made all data and (if applicable) computational code underlying the findings in their manuscript fully available?**

The PLOS Data policyrequires authors to make all data and code underlying the findings described in their manuscript fully available without restriction, with rare exception (please refer to the Data Availability Statement in the manuscript PDF file). The data and code should be provided as part of the manuscript or its supporting information, or deposited to a public repository. For example, in addition to summary statistics, the data points behind means, medians and variance measures should be available. If there are restrictions on publicly sharing data or code —e.g. participant privacy or use of data from a third party—those must be specified.

Reviewer #1: None

Reviewer #2: Yes

**********

PLOS authors have the option to publish the peer review history of their article (what does this mean?). If published, this will include your full peer review and any attached files.

If you choose “no”, your identity will remain anonymous but your review may still be made public.

**Do you want your identity to be public for this peer review?** For information about this choice, including consent withdrawal, please see our Privacy Policy.

Reviewer #1: No

Reviewer #2: No

[NOTE: If reviewer comments were submitted as an attachment file, they will be attached to this email and accessible via the submission site. Please log into your account, locate the manuscript record, and check for the action link "View Attachments". If this link does not appear, there are no attachment files.]

**Figure resubmission:**

**Reproducibility:**

?>

# Author comment: Author response to Decision Letter 1

**DOI:** 10.1371/journal.pcbi.1014818.r002

5 Jun 2026

Attachment Submitted filename: Response to Reviewers.docx

# Peer review: Decision Letter 1

**DOI:** 10.1371/journal.pcbi.1014818.r003

**Authors:** Hugues Berry

15 Jul 2026

How does explicit reliability guide choices? Effect of trustworthiness on choice and metacognition

PLOS Computational Biology

Dear Dr. Ota,

Thank you for submitting your manuscript to PLOS Computational Biology. After careful consideration, we feel that it has merit but does not fully meet PLOS Computational Biology's publication criteria as it currently stands. Therefore, we invite you to submit a revised version of the manuscript that addresses the points raised during the review process.

Please submit your revised manuscript by Sep 14 2026 11:59PM. If you will need more time than this to complete your revisions, please reply to this message or contact the journal office at ploscompbiol@plos.org. When you're ready to submit your revision, log on to https://www.editorialmanager.com/pcompbiol/ and select the 'Submissions Needing Revision' folder to locate your manuscript file.

Please include the following items when submitting your revised manuscript:

* A letter that responds to each point raised by the editor and reviewer(s). You should upload this letter as a separate file labeled 'Response to Reviewers'. This file does not need to include responses to formatting updates and technical items listed in the 'Journal Requirements' section below.

* A marked-up copy of your manuscript that highlights changes made to the original version. You should upload this as a separate file labeled 'Revised Manuscript with Track Changes'.

* An unmarked version of your revised paper without tracked changes. You should upload this as a separate file labeled 'Manuscript'.

Kind regards,

Hugues Berry

Section Editor

PLOS Computational Biology

Hugues Berry

Section Editor

PLOS Computational Biology

**Additional Editor Comments (if provided):**

**Reviewers' comments:**

Reviewer's Responses to Questions

**Comments to the Authors:**

**Please note here if the review is uploaded as an attachment.**

Reviewer #1: I'd like to thank the authors for addressing all my previous questions.

Reviewer #2: I have now carefully read the authors' replies to earlier comments, and I can see that the manuscript has strengthened.

There is one major point that remains not fully clarified is the computational model analysis, which I explain below (previous comment 4).

Supplementary Fig 12 indicates correlations between best fitting parameters are all relatively low, so the authors write that parameters are independent. In contrast, the authors elsewhere also write that parameters are not independent:

"The apparent S-shape may partly reflect modelling assumptions, because the sensitivity parameter cannot be estimated independently of the inverse temperature of the decision rule."

We are also told that the softmax temperature parameter is fixed not fitted, due to issues of identifiability; fixing this parameter is actually a strong assumption, which impacts the rest of the parameters interpretation for instance the weights. This assumption should be made more prominent, not only in the Methods section, for readers to properly interpret the findings.

The authors acknowledge in the Methods section that the other parameter observed differences could in principle reflect a difference in choice stochasticity, but then the whole paper does not shy away from interpreting the other parameter distortions and make conclusions on over/underweighting of information.

Model recovery: the authors have filtered out fits that did not converge. How many were filtered out besides the 64 fits selected? Could the authors explain how can we ensure that issues of convergence did not affect the fits of participants' data?

p108 the fitted weights are underestimated by the fitting procedure, not overestimated, as per fig supp 4

Next time, please print the quotes in the revised manuscript for each comment as is standardly done. It was time consuming to scroll back and forth to find edits in-between each response. In your final version please check the supplementary figures in the revised manuscript, many were mismatched with the legend or missing.

**********

**Have the authors made all data and (if applicable) computational code underlying the findings in their manuscript fully available?**

The PLOS Data policyrequires authors to make all data and code underlying the findings described in their manuscript fully available without restriction, with rare exception (please refer to the Data Availability Statement in the manuscript PDF file). The data and code should be provided as part of the manuscript or its supporting information, or deposited to a public repository. For example, in addition to summary statistics, the data points behind means, medians and variance measures should be available. If there are restrictions on publicly sharing data or code —e.g. participant privacy or use of data from a third party—those must be specified.

Reviewer #1: None

Reviewer #2: None

**********

PLOS authors have the option to publish the peer review history of their article (what does this mean?). If published, this will include your full peer review and any attached files.

If you choose “no”, your identity will remain anonymous but your review may still be made public.

**Do you want your identity to be public for this peer review?** For information about this choice, including consent withdrawal, please see our Privacy Policy.

Reviewer #1: No

Reviewer #2: No

[NOTE: If reviewer comments were submitted as an attachment file, they will be attached to this email and accessible via the submission site. Please log into your account, locate the manuscript record, and check for the action link "View Attachments". If this link does not appear, there are no attachment files.]

**Figure resubmission:**

**Reproducibility:**

?>

# Author comment: Author response to Decision Letter 2

**DOI:** 10.1371/journal.pcbi.1014818.r004

24 Aug 2026

Attachment Submitted filename: Response_to_Reviewers_auresp_2.docx

# Editor report: Decision Letter 2

**DOI:** 10.1371/journal.pcbi.1014818.r005

**Authors:** Hugues Berry

15 Sep 2026

Dear Dr. Ota,

We are pleased to inform you that your manuscript 'How does explicit reliability guide choices? Effect of trustworthiness on choice and metacognition' has been provisionally accepted for publication in PLOS Computational Biology.

Before your manuscript can be formally accepted you will need to complete some formatting changes, which you will receive in a follow up email. A member of our team will be in touch with a set of requests.

Please note that your manuscript will not be scheduled for publication until you have made the required changes, so a swift response is appreciated.

IMPORTANT: The editorial review process is now complete. PLOS will only permit corrections to spelling, formatting or significant scientific errors from this point onwards. Requests for major changes, or any which affect the scientific understanding of your work, will cause delays to the publication date of your manuscript.

Should you, your institution's press office or the journal office choose to press release your paper, you will automatically be opted out of early publication. We ask that you notify us now if you or your institution is planning to press release the article. All press must be co-ordinated with PLOS.

Thank you again for supporting Open Access publishing; we are looking forward to publishing your work in PLOS Computational Biology.

Best regards,

Hugues Berry

Section Editor

PLOS Computational Biology

Hugues Berry

Section Editor

PLOS Computational Biology

***********************************************************

# Editor report: Acceptance letter

**DOI:** 10.1371/journal.pcbi.1014818.r006

**Authors:** Hugues Berry

PCOMPBIOL-D-25-02202R2

How does explicit reliability guide choices? Effect of trustworthiness on choice and metacognition

Dear Dr Ota,

I am pleased to inform you that your manuscript has been formally accepted for publication in PLOS Computational Biology. Your manuscript is now with our production department and you will be notified of the publication date in due course.

The corresponding author will soon be receiving a typeset proof for review, to ensure errors have not been introduced during production. Please review the PDF proof of your manuscript carefully, as this is the last chance to correct any errors. Please note that major changes, or those which affect the scientific understanding of the work, will likely cause delays to the publication date of your manuscript.

Soon after your final files are uploaded, unless you have opted out, the early version of your manuscript will be published online. The date of the early version will be your article's publication date. The final article will be published to the same URL, and all versions of the paper will be accessible to readers.

For Research, Software, and Methods articles, you will receive an invoice from PLOS for your publication fee after your manuscript has reached the completed accept phase. If you receive an email requesting payment before acceptance or for any other service, this may be a phishing scheme. Learn how to identify phishing emails and protect your accounts at https://explore.plos.org/phishing.

Thank you again for supporting PLOS Computational Biology and open-access publishing. We are looking forward to publishing your work!

With kind regards,

Janani Seenivasan

PLOS Computational Biology | Carlyle House, Carlyle Road, Cambridge CB4 3DN | United Kingdom ploscompbiol@plos.org | Phone +44 (0) 1223-442824 | ploscompbiol.org | @PLOSCompBiol
